# Laboratory Models for Investigating Breast Cancer Therapy Resistance and Metastasis

**DOI:** 10.3389/fonc.2021.645698

**Published:** 2021-03-10

**Authors:** Kevin Roarty, Gloria V. Echeverria

**Affiliations:** ^1^Department of Molecular and Cellular Biology, Baylor College of Medicine, Houston, TX, United States; ^2^Dan L. Duncan Cancer Center, Baylor College of Medicine, Houston, TX, United States; ^3^Lester and Sue Smith Breast Center, Baylor College of Medicine, Houston, TX, United States; ^4^Department of Medicine, Baylor College of Medicine, Houston, TX, United States

**Keywords:** breast cancer, metastasis, chemoresistance, genetically engineered mouse models, patient derived xenograft (PDX) model, cancer cell lines

## Abstract

While numerous therapies are highly efficacious in early-stage breast cancers and in particular subsets of breast cancers, therapeutic resistance and metastasis unfortunately arise in many patients. In many cases, tumors that are resistant to standard of care therapies, as well as tumors that have metastasized, are treatable but incurable with existing clinical strategies. Both therapy resistance and metastasis are multi-step processes during which tumor cells must overcome diverse environmental and selective hurdles. Mechanisms by which tumor cells achieve this are numerous and include acquisition of invasive and migratory capabilities, cell-intrinsic genetic and/or epigenetic adaptations, clonal selection, immune evasion, interactions with stromal cells, entering a state of dormancy or senescence, and maintaining self-renewal capacity. To overcome therapy resistance and metastasis in breast cancer, the ability to effectively model each of these mechanisms in the laboratory is essential. Herein we review historic and the current state-of-the-art laboratory model systems and experimental approaches used to investigate breast cancer metastasis and resistance to standard of care therapeutics. While each model system has inherent limitations, they have provided invaluable insights, many of which have translated into regimens undergoing clinical evaluation. We will discuss the limitations and advantages of a variety of model systems that have been used to investigate breast cancer metastasis and therapy resistance and outline potential strategies to improve experimental modeling to further our knowledge of these processes, which will be crucial for the continued development of effective breast cancer treatments.

## Introduction

### Breast Cancer Metastasis and Therapy Resistance

Breast cancer is the most commonly diagnosed cancer in women and results in 40,000 deaths in the United States annually. The presence of hormone receptors (HR), specifically estrogen receptor (ER) and progesterone receptor (PR), together with expression and amplification of the human epidermal growth factor receptor type 2 (HER2), help to broadly classify breast cancer into three main clinical subtypes: ER/PR+, HER2+, or triple negative (TNBC). The histological classification of breast cancer by HR status largely dictates treatment decisions today. However, the molecular stratification of breast cancer, discovered almost 20 years ago by cDNA microarray of breast tumors, further unmasked intrinsic subtypes of breast cancer. These molecular portraits of breast cancer, now based on a 50-gene classifier (PAM50) (1, 2), help depict the molecular heterogeneity both within and across these intrinsic subtypes and offer a molecular complement to histological classifications. Although standard of care (SOC) therapeutic regimens vary amongst the major breast cancer subtypes, therapy resistance and metastasis remain shared clinical issues for all types of breast cancer.

Metastatic breast cancer accounts for the vast majority of breast cancer related deaths. However, our understanding of this process is still largely evolving. Further complicating this multistep process is the inherent heterogeneity present within a patient’s tumor (intra-tumor heterogeneity) and the fact that the intrinsic classification of breast cancer shapes both the timing and location of metastatic relapse. For instance, while HR+ breast cancers tend to home to the bone and lymph nodes, TNBCs exhibit a preference for visceral organs like the lungs, liver, and brain. HER2+ breast cancers tend to metastasize to the brain after averting HER2-targeted therapies. Additionally, the timing of metastatic presentation also differs by breast cancer subtype, with HR+ tumors typically recurring later than TNBCs after initial presentation and treatment of the primary disease. The predilection of subtypes to home to specific locations in the body, the subtype-dependent variation in recurrence windows, as well as how particular subpopulations of tumor cells within these cancers accomplish metastatic steps remain imperative questions to answer in order to mitigate breast cancer mortality.

Despite considerable appreciation for the subtype-specific differences in timing and location of metastatic disease, knowledge is lacking on how to accurately predict a tumor’s metastatic fitness as well as the specific biological mechanisms instructing the stage-specific steps of the metastatic cascade. The development of *in vitro* and *in vivo* models over several decades has helped illuminate the metastatic process. Considerable work remains to improve such models in order to gain molecular insights into metastasis and therapeutic resistance, the primary culprits of cancer-related deaths.

### Laboratory Models of Breast Cancer

**Figure 1:**
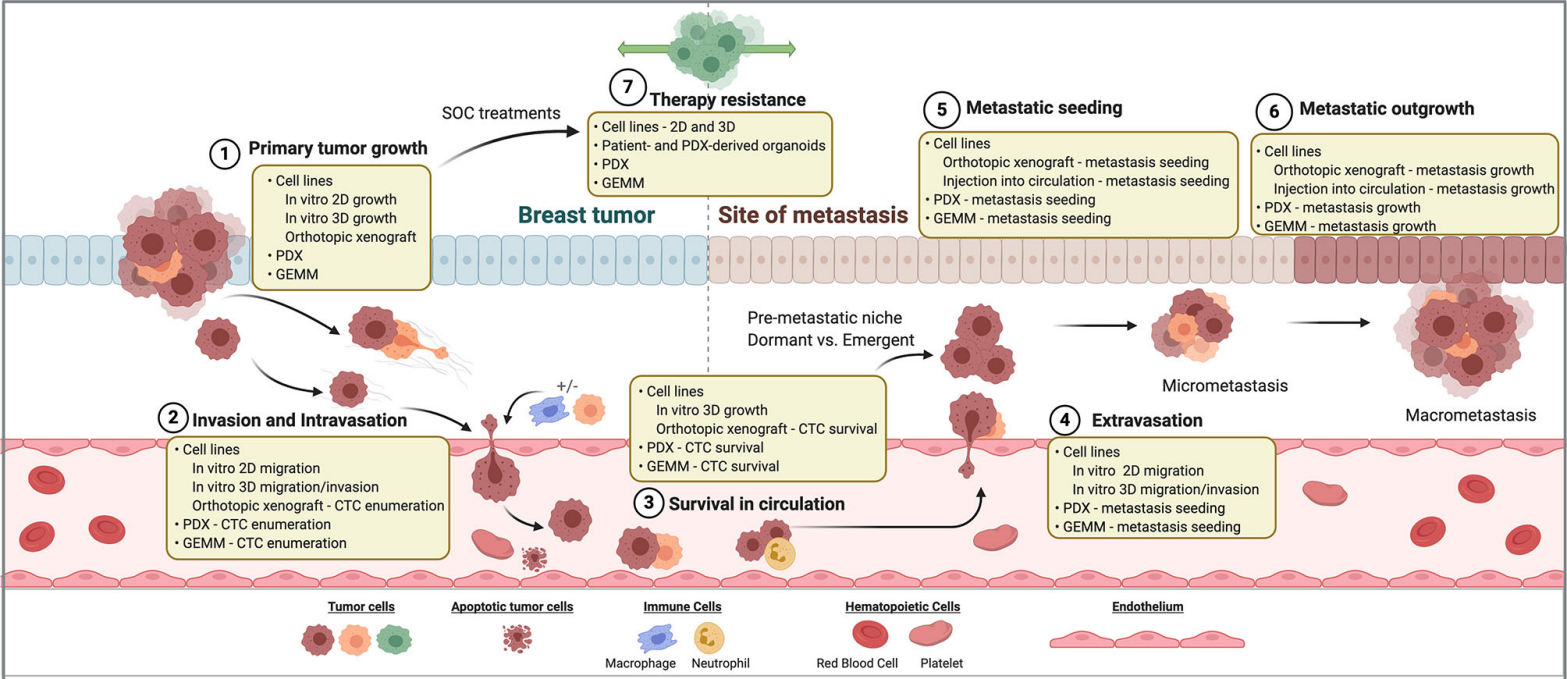
Breast cancer models for investigating therapy resistance and metastasis. Steps of the metastatic cascade and SOC therapy resistance are diagrammed. For each step, classes of laboratory models that may be used to investigate its biology are listed. SOC, standard of care. PDX, patient-derived xenograft. GEMM, genetically engineered mouse model. CTC, circulating tumor cell.

Metastasis is a multistep process that requires the successful dissemination of tumor cells from the primary site, vascular entry (intravasation) and transit to a distant site, exit (extravasation) from the vasculature into the secondary site, and finally seeding and colonization in the secondary organ site. Importantly, the accomplishment of only one phase of the metastatic cascade by the tumor cell does not necessarily predict successful fulfillment of metastasis as a whole. Thus, experimental models and interpretation of the mechanisms derived from these models is imperative in order to differentiate successful from unsuccessful metastasis and the consequential events dictating a tumor cell’s fitness to evade, spread, and thrive a distant site from the breast. The multistep nature of metastasis and the heterogeneity exhibited within breast cancer warrants the continued use and development of laboratory models to accurately reflect this complicated process in order to discover therapeutic interventions. To date, a compilation of experimental models has shed light on mechanisms surrounding invasion and dissemination, tumor cell dormancy, organ tropism, and microenvironment interactions (**Figure 1**). How these biological events are shaped by therapeutic interventions adds another level of complexity surrounding metastasis and disease recurrence.

Mechanisms of therapy resistance in breast cancer are diverse amongst breast cancer subtypes and mechanism of action of each therapy. Mechanisms of therapy resistance have been found to be particularly different in the cases of molecularly targeted versus cytotoxic chemotherapies. Therapeutic resistance can be intrinsic, or pre-existing in tumors prior to drug exposure, or acquired following drug treatment. Both intrinsic and acquired resistance can be achieved through clonal evolution (*de novo* acquisition of mutations or genomic structural changes), clonal dynamics (enrichment and/or depletion of genomic subclones through Darwinian selection), epigenetic adaptations (chromatin modification, transcriptional and post-transcriptional cellular plasticity, microenvironmental crosstalk, metabolic regulation), and acquisition or maintenance of cancer stem-like cell (CSC) features. While some genomic mechanisms of therapy resistance have been appreciated for decades, models to study epigenetic-mediated mechanisms of resistance have been developed more recently. As an added layer of complexity, many non-genomic resistance mechanisms have been found to be reversible, such as drug tolerant or persister cell states. Thus, elucidating the temporal nature of resistance mechanisms is of utmost importance to effectively identify appropriate therapeutic windows. Laboratory models to investigate these complex mechanisms will be discussed below (**Figure 1**).

## Models of Metastasis

The establishment of distant metastasis necessitates the cancer cells to overcome several key hurdles along the journey from the primary tumor to a distant organ. Numerous *in vitro* and *in vivo* models have enabled the exploration of mechanisms surrounding the various steps of metastasis, yet the accurate recapitulation of the multi-step process of the metastatic cascade varies drastically from model to model. Though metastasis is traditionally viewed as a linear series of events, often accomplished by the fittest of cancer cells (3), numerous questions remain surrounding not only the mechanisms governing these discrete steps, but also concepts surrounding dormancy and the emergence of metastatic lesions after months to years. The metastatic cascade can also be impacted by somatic mutation-driven mechanisms. For example, numerous ESR1 mutations and gene fusions have been identified in metastatic or liquid biopsies from ER+ breast cancer patients. Introduction of many of these mutations into *in vitro* and *in vivo* laboratory models (some even naturally occur in patient-derived xenografts, PDXs) has enabled demonstration that they functionally drive metastasis through aberrant ESR1 signaling (4–7). Further description of these mutations can be found in our discussion of therapy resistance in ER+ breast cancer. On the other hand, the metastatic cascade can also be driven by non-genetic (*i.e.* epigenetic) mechanisms that can be modeled in the laboratory, such as tumor cell-microenvironmental interactions. The continued mystery surrounding multiple facets of breast cancer metastasis and the need to develop therapies around this advanced stage of disease requires a renewed approach by investigators to develop and use models with increased physiological relevance, whether *in vitro* or *in vivo*. Specifically, how experimental models accurately reflect early versus late recurrences, distinguish metastatic risk among patients, and provide an accurate approximation of the metastatic process that can be extrapolated to patients remain imperative questions to answer. The model platforms, as well as the advantages and disadvantages of various systems, will be summarized in the current section (**Table 1**).

**Table 1:** Benefits and drawbacks of laboratory models to study breast cancer therapy resistance and metastasis.

Application	Type of Model	Advantages	Disadvantages
Metastasis	*In vitro* – 2D	Ease of experimental and genetic manipulation Precise control of variables Ability for longitudinal/kinetic measurements Several established assays for 2D migration Potential for scalability	Lack of microenvironment Lack of complete ECM complement Lack of biophysical forces and barriers to invasion Genetic drift due to long-term culture Inability to recapitulate complete heterogeneity of patient tumors
	*In vitro* – 3D	Closer physiological relevance to primary tumor Numerous established assays for 3D invasion/migration/ECM interactions Increased control of experimental inputs (cellular and ECM composition) Ability to visualize heterotypic or cell-matrix interactions Potential for scalability Better predictors of *in vivo* drug responses compared to 2D Low cost to analyze patient tumor cells compared to PDX establishment	Not all tumor samples can survive *in vitro*, restricting experiments to short-term cultures Lack of complete *in vivo* microenvironment
	*In vivo* – injection into circulation	Ability to model later stages of the metastatic cascade Site-specific development of metastasis Opportunity to utilize several tumor models and cell lines Immunocompetent if syngeneic line used Readily reproducible	Inability to model early stages of the metastatic cascade Immunocompromised host if material is PDX- or human cell line-derived
	*In vivo* – orthotopic xenografts	Ability to model the entire metastatic cascade Ability to experiment with minimally manipulated human tumor biopsies Intact mammary microenvironment Degree of genetic and phenotypic intratumor heterogeneity closely matches patients Ability to experiment with minimally manipulated human tumor biopsies Ability to obtain multi-site metastases Ability to transduce and label tumor cells for metastasis studies Ease of separating tumor from stroma based on species	Deficient immune system Mouse, not human, microenvironment High cost Lengthy time for tumor establishment and passaging The majority of patient tumors will not engraft as PDXs – TNBC advantage over ER+ in engraftment
	*In vivo* – GEMMs and syngeneic transplants	Ability to model the entire metastatic cascade Intact immune system and complete microenvironment Some degree of genetic and phenotypic heterogeneity Ability to genetically control metastasis Relative low cost of animal purchase compared to PDXs	Tumors are initiated by only a few oncogenic events over a relatively short time scale Lack of models of ER+ breast cancer metastasis Organ tropism not always reflective of clinical setting Sometimes long timescales of tumorigenesis Necessitates genetic breeding colony
Therapy resistance	*In vitro* – 2D	Same as above Ability for longitudinal monitoring of resistance dynamics and reversibility Ease of testing large-scale drug combinations Numerous established assays for drug efflux, CSC features, cell survival/viability Ability for longitudinal monitoring of resistance dynamics and reversibility	Same as above
	*In vitro* – 3D	Same as above Have been demonstrated to recapitulate heterogeneity and epigenetic features of patients’ tumors Relative ease of testing large-scale drug combinations Ability for longitudinal monitoring of resistance dynamics and reversibility	Same as above Drug screening assays must be amenable to 3D viability or morphological readouts
	*In vivo* - PDXs	Same as above Ability to study drug pharmacokinetic/distribution properties in a whole organism Ability to test novel agents in ‘preclinical trials’ comparable to human clinical trials Ability to serially expand therapy resistant tumors Ability for longitudinal monitoring of resistance dynamics and reversibility	Same as above Inability to fully evaluate the efficacy of therapies that are modulated by the immune system
	*In vivo* - GEMMs	Same as above Ability for analysis of genetic drivers of resistance Ability to serially expand therapy resistant tumors Ability to test stroma-targeted therapies Ability for longitudinal monitoring of resistance dynamics and reversibility	Same as above Inability to fully model the impacts of therapy on intratumor heterogeneity

### *In Vitro* Models of Metastasis—2D

*In vitro* models encompass a variety of assays of different structural, microenvironmental, and cellular composition that provide controlled experimental systems to extrapolate cellular processes implicated in the metastatic cascade. Given the elusive biology of metastasis *in vivo, in vitro* models offer a surrogate approach to interrogate mechanisms responsible for fulfilling discrete steps in the metastatic cascade. Typically, these approaches have been instrumental to examine the functional implications of a particular gene or pathway in metastasis and provide a defined platform to quantitatively assess cell function associated with cell proliferation, survival, invasion, adhesion, and cell–cell and -microenvironment interactions. Additionally, the adoption of more heterogenous cell models through genetically engineered mouse models (GEMMs), PDXs, or primary cells directly from patients for *in vitro* studies has the potential to significantly enhance our understanding of metastasis.

The initial steps of metastasis require that tumor cells disseminate or invade from the breast. This initial step of metastasis requires that cells gain migration capacity. The scratch or wound healing assay is one such *in vitro* assay in a two-dimensional (2D) space that measures the ability of a monolayer of tumor cells to fill a “wounded” area created experimentally by introducing a scratch through the cell sheet. Often these assays are applied to studies that query the function of a particular gene in the regulation of migration properties. The application of live-cell microscopy can provide a level of quantitation that enables the establishment of cell migration kinetics over time. Despite the relative ease of the 2D invasion assay, the scalable nature of the method, and the relative flexibility of the system with multiple cell inputs, these 2D cell models differ considerably from *in vivo* models. Namely, their spatial organization, cell interactions, and intercellular signaling can differ substantially from the physiologically complex three-dimensional (3D) space of a tumor. Indeed, drug screening outcomes in 2D systems often fail to accurately recapitulate the *in vivo* setting (8, 9).

Cancer cell invasion and dissemination often involve chemotaxis, the directed movement of cells by an extracellular gradient. Boyden chamber assays enable the experimental evaluation of these phenomena by the seeding of cells on an upper chamber and monitoring the migration of cells through a defined porous membrane toward a chemoattractant in the bottom well. Given the separation of migrating vs. refractory cells on the bottom and upper chambers, respectively, migration-competent cells can be recovered and evaluated in response to a particular chemical or physical gradient in an effort to identify subpopulations with potentially distinct invasive potentials. These approaches helped establish the bone-tropic mouse mammary 4T1 carcinoma cells from repeated chemotactic selection *in vitro (*10). Adaptations of the Boyden chamber have evolved to include additional matrices and cell types to enable the evaluation of other metastatic steps, such as intravasation and extravasation. The modified Boyden chamber assay, for example, includes Matrigel, fibronectin, or collagen I to the trans-well porous membrane in order to model the extracellular matrix (ECM), a critical component in cellular migration. The addition of macrophages and endothelial cells to such a modified trans-well system, termed the subluminal to luminal trans-endothelial migration assay (iTEM), identified the presence of macrophages as an important niche factor for invasive tumor cells highly expressing an actin regulatory protein, Mena^INV^, to traverse the endothelium during intravasation (11–13). The plating of endothelial cells within this system provided an additional component that enabled the evaluation of invasion through cell-cell junctions of the endothelium and the ECM.

### *In Vitro* Models of Metastasis—3D

3D models have gained considerable attention lately to better recapitulate the multicellular interactions of tumor cells within a defined ECM. These models can be generated from GEMMs, breast cancer cell lines, PDX tumors, or tumors obtained directly from breast cancer patients. In contrast to 2D in vitro systems, 3D approaches provide a platform to study cellular heterogeneity, cellular plasticity, cell-cell, and cell-ECM interactions and have evolved to provide a more physiologically relevant *in vitro* platform to interrogate the metastatic program (**Table 1**). Since the advent of organoid cultures for the investigation of cell organization and polarity in 3D basement membrane contexts, molecular insights into the heterogeneity of the primary tumor now demand the adaptation of the 3D system to accurately reflect the level of complexity *in vivo*. 3D organoid biobanks have emerged as a comprehensive representation of the phenotypic and molecular heterogeneity from patient tumors (14–16). In addition to GEMM and cell line models, they represent an extremely powerful resource for ongoing development of engineered 3D systems as models for metastasis and therapeutic resistance.

3D systems rely on the ECM, known to be intricately involved in breast cancer metastasis. The ECM of both primary tumor and distant metastatic sites are composed of insoluble proteins (*e.g.*, collagen, laminin, fibronectin, and elastin), glycosaminoglycans, and proteogIycans. In particular, the deposition, remodeling, and crosslinking of ECM within the primary tumor regulates both mechanical and biochemical cues for the cancer cells, and “stiffer” tumors often exhibit poorer prognosis (17). Multiple 3D organoid models have implicated matrix composition as a critical regulator of tumor cell transit. For instance, the mode of migration by carcinoma cells, specifically single or collective in nature, is impacted by the presence of Type 1 collagen, independent of the genetic state of the tumor cell (18). Similarly, the conserved cytokeratin 14 (K14^+^) basal epithelial program orchestrates collective leader-follower cell behaviors during tumor cell invasion in 3D Type 1 Collagen (19). Friedl and colleagues demonstrated that leader cell function depends on a gap junction Cx43-dependent/ADORA1 axis in mediating collective cancer cell invasion (20). Interestingly, cadherins and ECM confinement further cooperate to determine unjamming transitions, coordinated vs. uncoordinated collective cell movements, and fluidization of tumor cells, impacting states of cell transit at matrix bottlenecks (21). Introduction of microfluidic systems by soft lithography techniques to such organoid models further revealed the importance of a chemotactic SDF1/CXCR4 gradient necessary for positioning K14^+^ leader cells within invasive cellular collectives (22).

While the above 3D organoid models largely focus on mechanisms of tumor cell invasion within the ECM, organotypic cultures have recently evolved in their level of sophistication to address biological questions related to additional stages within the metastatic cascade. For instance, immune cell introduction into 3D organoid models of invasion addresses the immunosurveillance bottleneck encountered by tumor cells, revealing important functions for natural killer cell and tumor cell crosstalk on the invasion of K14^+^ cells (23). Reconstitution of 3D cultures of established breast cancer cell lines with immune cells offers additional models to interrogate immune- and tumor-cell interactions *in vitro* (24). Organotypic models have more recently been developed to model the metastatic niche, where questions of tumor cell dormancy and colonization can be addressed. For instance, *in vitro* co-cultures of organotypic microvascular niches and disseminated tumor cells (DTCs) identified the importance of the microvascular niche in distinguishing states of tumor cell dormancy versus emergence based on thrombospondin-1 and TGF-β availability (25). Additional complex organotypic cultures, such as the Bone-In-Culture-Array (BICA) have been developed to determine mechanisms of early-stage bone colonization (26, 27).

Organotypic cultures of the metastatic niche provide an important platform for drug screening. For instance, BICA revealed the utility of danusertib, an Aurora kinase family inhibitor, as a potential therapeutic inhibiting early-stage bone colonization (26). Moreover, DTCs were protected from chemotherapy by an α5β3 and α4β1 integrin-mediated interaction with the perivascular niche (28). Using organotypic cultures, integrin inhibitors disrupted this protection and rendered DTCs susceptible to chemotherapy. Thus, tailored drug screening using organotypic cultures of breast cancer cells and cells of the microenvironment offer more high-throughput and less costly alternatives to therapeutic testing *in vivo*.

### *In Vivo* Experimental Models of Metastasis

Experimental metastasis refers to the introduction of tumors cells directly into the vascular system, circumventing the early stages of the metastatic cascade. This approach has been useful to explore the functional roles of distinct genes in metastatic colonization and to test therapeutic agents in late-stage metastasis. Importantly, experimental models of metastasis simulate extravasation and colonization in the secondary site, reflecting later stages of disease, as opposed to spontaneous models (described below), which model the full extent of the metastatic cascade. In the case of PDXs and human cell lines, the majority of these injection studies are conducted in immunodeficient mice, precluding analysis of the immune system. Despite this limited snapshot of the metastatic process, the application of such an approach by Fidler and colleagues sparked the landmark discovery that only subpopulations of cells possess metastatic abilities, and these could be clonally selected to derive lines with enhanced metastatic seeding to a particular organ (29).

Importantly, experimental models of metastasis are largely dictated by the site of injection and inherent tropism of the tumor cells. Although these studies rely heavily on the lodging of tumor cells into the first capillary bed encountered downstream of the location of vascular delivery, mechanisms of Paget’s seed-and-soil hypothesis have been pursued to identify factors involved in organ-specific metastasis (3). For instance, lateral tail vein injections largely result in pulmonary metastases (30), intracardiac injections prompt metastasis in the bone and brain (31), intracarotid injection similarly route to the brain, and intra-iliac artery injections selectively seed bone metastasis (32). Using such approaches, studies were performed to identify genes that orchestrate breast cancer metastasis to specific organs. One widely used model, the lung-tropic MDA-MB-231 LM2 cells, was derived by selection of a subline from the parental MDA-MB-231 TNBC cells with greater metastatic proclivity to the lungs (30). Similar bone-tropic (33) and brain-tropic (31) sublines of MDA-MB-231 cells were also derived using similar methodologies. Experimental metastasis models have been instrumental to establish metastatic derivatives of other human and mouse breast cancer cell lines, such as MCF7 (34), 4T1 (35), and T47D (36). Thus, collective efforts over the years have leveraged the experimental metastasis model and the utility of such a model to dissect mechanisms of extravasation and tumor cell colonization. While noteworthy, these studies exclude earlier stages of metastasis, limiting the full physiological comparison to appropriately model aspects of the selective pressures encountered by tumor cells within the earlier stages of the metastatic cascade, the potential interclonal tumor cell interactions required throughout the metastatic process, additional tumor-host cell interactions during transit, and the elusive biology surrounding tumor cell dormancy. Despite these limitations, experimental models of metastasis have provided a reproducible approach to interrogate aspects of metastatic fitness. Recently, a sophisticated strategy involving lentiviral barcoding and scaling across several human basal-like cell lines as proof-of-principle used pan-cancer PRISM cell line pools for high-throughput metastatic potential mapping (37). Using this approach, an altered lipid metabolism state was associated with brain metastasis in basal-like breast cancer. Though this pan-cancer “MetMap” lacked the context of an intact immune system, such a study provides a valuable resource to probe metastatic potential across tumor types.

### *In Vivo* Orthotopic Xenograft Models of Metastasis

A major advantage of orthotopic models of breast cancer metastasis, in which breast cancer cells are engrafted into the mammary glands of mice, is that they capture all steps of the metastatic cascade. These models enable direct comparison of primary tumors, circulating tumor cells (CTCs), and metastases matched within the same animal. Importantly, some models metastasize to multiple secondary sites, enabling comparisons of tumor cells growing in distinct secondary organ microenvironments. Numerous breast cancer cell lines have been orthotopically xenografted into mice for CTC and metastasis studies (33, 38, 39). PDX models, in which never-cultured biopsies are obtained from patients and directly engrafted into mice, have been found to capture molecular features and heterogeneity of originating patients’ tumors and serve as a renewable resource of minimally manipulated human tumor cells (16, 40–42). The primary disadvantages of these models are: 1) the requirement of using immune-compromised mice, thus precluding assessment of the impact of a fully intact immune system on metastasis, 2) the often-lengthy duration of experiments, regularly up to 12 months, and 3) the costly nature of immune-compromised animal purchase and long-term housing. A major need in the field is the broad implementation of xenograft models in mice with ‘humanized’ immune systems.

Ideally, PDXs should reflect the full range of cellular heterogeneity and disease progression across breast cancers. The PDX consortium, a shared effort comprised of several academic institutions, has amassed 537 PDX lines representing 500 patients (40). An open question remains regarding how accurately these PDXs reproduce the metastatic behavior of the patient’s tumor, as well as more general metastatic characteristics associated with breast cancer subtype. Although considerable evidence exists that these PDXs can produce CTCs and generate micro- and macroscopic metastatic lesions within several distant sits in the mouse (41, 43–45), a full credentialization of the metastatic propensity of this vast tissue resource remains an evolving collective task. Given that ER+ cancers typically exhibit longer latency and a proclivity to metastasize to bone, the development of humanized mouse models in which breast cancer PDXs metastasize to human bone implants has created a highly reliable system to interrogate late-stage metastasis to the bone (46). Specifically, bone discs from femoral heads of patients undergoing hip replacement surgery were implanted subcutaneously into NOD/SCID mice. This model system resembles a prior human-in-mouse bone system where breast cancer cell lines, instead of PDXs, were used (47). Nonetheless, human bone was the preferred site of metastasis for ER+ PDXs over mouse bone, while TNBC PDXs metastasized at a lower rate to bone, but with an increased frequency of visceral metastasis. Thus, PDX models can accurately recapitulate site-specific preferences of metastasis for breast cancer subtypes.

Spontaneously arising metastases in PDX models, sometimes even to distinct secondary organs, enable powerful comparisons that are usually impossible in the clinical setting due to limited availability of metastatic specimens. PDX models have been found to faithfully recapitulate secondary organ tropisms of their originating patient tumor (40, 41, 43). A major benefit of PDX models is the difference in species between the tumor and stromal compartments, enabling relative ease of separating these in the laboratory and informatically. While markers universally recognizing human tumor cells are uncommon, human CD298 has been used with success to isolate viable human tumor cells from early- and late-stage PDX mammary tumors and lung metastases (48, 49). Obtaining macroscopic metastatic lesions from PDX models, especially in secondary organ sites aside from the lung, is extremely uncommon. Incorporating survival surgery, in which mammary tumors are grown nearing ethical tumor burden endpoints, then resected, enables monitoring of mice for longer periods to allow detectable metastatic lesions to arise. This approach has been used with success in several PDX models, some of which metastasize robustly to multiple secondary organs. This methodology is majorly bolstered by incorporation of *in vivo* imaging constructs (*e.g.* bioluminescent markers), allowing *in vivo* and *ex vivo* detection of metastatic lesions from multiple secondary organs of the mouse (50, 51). These models have also enabled comparison of tumor cell subpopulations growing as primary tumors, CTCs, and metastatic lesions. In particular, *in vivo* modeling of CTC tumor cell biology to capture vascular transit has been demonstrated directly from patient blood specimens together with *in vivo* validation in cell line xenografts. The differential labeling of the MDA-MB-231 LM2 cell line with eGFP and mCherry fluorescence enabled the detection of multicolor CTC clusters in circulation, which were later shown to be oligoclonal precursors of metastasis to the lung requiring plakoglobin for collective tumor cell transit (52). Interestingly, such CTC collectives preferentially arose in hypoxic areas of the tumor, as demonstrated in patient and cell line specimens (53). Similar studies using MDA-MB-231 or murine 4T1 cell lines further demonstrated the requirement of neutrophils to facilitate CTC cluster cell cycle entry, heightening metastatic conditioning in the circulation (54). While powerful, extrapolation of such approaches to cell line or PDX models necessitates prior introduction of lentiviral or alternative cell labels for accurate tracking and identification of rare cell populations *in vivo*.

### Enrichment and Screening of Metastasis With *In Vivo* Xenograft Models

To identify genes suppressing colonization of the lung, a high-throughput RNAi screen of ~1,000 genes was conducted by intravenously injecting pools of mouse mammary tumor 4T1 cells expressing siRNA constructs into Balb/c mice (55). Bioluminescence imaging was used to quantify lung colonization for each of 48 pools, and next-generation sequencing was used to identify siRNAs enriched in lung lesions. This screen identified alpha-N-acetylgalactosaminide alpha-2,6-sialyltransferase 2 (St6GalNAc2) as a novel metastasis suppressor that acts through its O-glycanation of the surface of tumor cells. A major advantage of this approach is use of immune-competent Balb/c mice. While this screen focused on the final steps of the metastatic cascade (colonization and outgrowth in the secondary organ site), additional screens encompassing the entire metastatic cascade from the orthotopic site are warranted in order to piece together mediators of specific phases of metastasis. Genetic screens focused on the regulation of CTCs have shed light on important regulators of CTC composition and function during vascular transit. One such screen entailed a CRISPR-Cas9 loss-of-function mini-pool screen *in vivo* to evaluate guide RNA dropouts, with Vcam1 identified as a necessary factor for CTC-neutrophil interactions (54). Additionally, an *in vivo* genome-wide CRISPR activation screen was performed on CTCs to screen for pro-metastatic genes. Together with single cell RNA sequencing from patient CTC specimens, Rpl15-dependent ribosomal protein upregulation was implicated in proliferative and survival cues for CTCs *in vivo* (56).

Orthotopic xenograft models have been a rich model system with which to conduct *in vivo* functional genomics screens for genes driving or suppressing metastasis. A recent study employed TNBC PDX tumor cells transduced with an ORF library orthotopically injected into mice, then utilized bioluminescence imaging to obtain lung metastases. Genes decreasing lung metastasis latency were then identified by next generation sequencing of lung lesions (57). This custom ORF library was constructed to over-express genes identified from differential expression analysis of human genes identified by RNA sequencing of lung metastases and matched mammary tumors from PDX models and successfully identified a validated driver of breast cancer metastasis, CEACAM5, that is currently under clinical investigation. While *in vivo* metastasis screens are arguably one of the most powerful approaches available to identify genes with a *bona fide* function in the metastatic cascade, these screens are costly and, especially in the case of orthotopic xenografts, can require long periods of time. Thus, focusing such screens on a prioritized subset of genes is critical to minimize the cost and scale of this undertaking.

### Genetically Engineered Mouse Models and Syngeneic *In Vivo* Transplant Models of Metastasis

A considerable number of GEMMs exist that utilize constitutive or inducible transgenic approaches to model tumor progression and metastasis. By far the most widely used system is the Mouse Mammary Tumor Virus (MMTV) LTR promoter, among several other promoters (WAP, BLG, and C- (3)1) (58), that has been used to readily drive the expression of transgenes specifically in the mammary epithelium. Key oncogenes explored within the mammary epithelium include ErbB2/Neu (59), polyoma middle T antigen (PyMT) (60), Simian virus 40 (SV40) (61), Wnt1 (62), TGF-α (63), c-Myc (64), and H-Ras (58). MMTV-Neu and MMTV-PyMT represent two of the most well-characterized transgenic mouse models of mammary tumorigenesis, which readily metastasize to the lung, albeit at different rates (58).

By far, the most widely utilized models over the past 20 years include the MMTV-Neu and MMTV-PyMT models. MMTV-neu transgenic mice develop multifocal mammary tumors at a median age of 7.5 months and metastasize to the lungs (65–67). MMTV-PyMT mice, on the other hand, metastasize with higher frequency and shorter latency (60). Recent integrative genomic analyses of both models identified critical parallels with human breast cancers, particularly copy number alterations in key ECM and other proteins that drive metastasis in these models (68). Over the years, both models were instrumental in establishing the biological functions for the TGF-β (69–72), Wnt (73), and EGF (72) pathways in breast cancer progression and metastasis. Importantly, these models incorporated the thorough examination of endogenous tumor–stroma interactions associated with metastatic progression (74). As genetic and technological advances developed, higher resolution cell biology and live microscopy approaches unveiled previously furtive cellular interactions occurring along the metastatic cascade. Findings from such studies unveiled important tumor cell-macrophage interactions critical for vascular leakage and intravasation (75–77). MMTV-PyMT transgenic mice were also utilized to uncover collective tumor cell interactions during invasion, ultimately responsible for oligoclonal metastasis (78). Follow-up studies further implicated nanolumenal signaling between tumor cell clusters via the molecule epigen during oligoclonal metastasis (79). To more accurately depict breast cancer subtype, the *TP53*-null syngeneic transplant model of mammary tumorigenesis comprises a biobank of tumors that reflect heterogeneity of human breast cancers at the molecular and histological levels (80–82). Importantly, the *TP53*-null syngeneic transplantable GEMM harbors an intact immune system, which has been an instrumental modulator of metastatic propensity to the lung (83). Given the molecular and histological representation of cellular heterogeneity, this transplant model has enabled the study of various aspects of the metastasis and therapeutic resistance (83, 84) Establishment of organ-tropic models from this heterogeneous GEMM will provide an invaluable resource to study the contributions of inter- and intra-tumor heterogeneity (Roarty, unpublished). The foremost advantage of these GEMMs is the ability to experimentally probe the entirety of the metastatic cascade in the context of an intact immune system.

Spontaneous models of metastasis also hold great promise to unravel mechanisms of tumor cell dormancy in the metastatic niche. A persistent mystery in cancer biology is the “lag” or emergence of metastasis several months, years, or decades following removal of the patient’s primary tumor. Although it is appreciated that the time-to-relapse and cancer cell tropism exhibited in breast cancer are dictated largely by the intrinsic subtype of the tumor (85), the exact timing of dissemination during cancer progression and how such fleeing cells later emerge as metastatic lesions remains unknown. Several lines of evidence demonstrate a lack of linearity in the metastatic process. In patients, disseminated tumor cells in the bone marrow were found to harbor fewer genetic alterations than the primary lesion, suggesting that these precursors arose earlier rather than later in advanced stages of disease progression (86). Mouse models have molecularly exposed this lack of linearity seen in humans (87), where non-invasive mammary intraepithelial neoplasia (MIN), arising in both MMTV-neu and MMTV-PyMT transgenic models, were capable of releasing disseminated cells into the circulation of mice, leading to micrometastasis within the bone marrow and lungs (88). Such early disseminated cancer cells can fulfill all steps of metastasis, as has been demonstrated in the MMTV-neu model, where Wnt signaling and a hybrid EMT-dependent program enable metastasis after a period of dormancy (89). The switch from dormant to active metastatic states is an ongoing area of investigation, but one that is yielding interesting findings of the constant interplay between cancer cells and their extracellular and immune microenvironment in this process (25, 90–94). Thus, the utility of mouse models to interrogate the molecular regulation of dormant versus active metastatic states will be an imperative endeavor to provide important therapeutic insights.

The recent success of immune checkpoint inhibitors (ICIs) in improving patient outcomes has only amplified a growing interest the application of such therapies to breast cancer (95). Syngeneic models of metastasis offer a unique opportunity to interrogate the immune landscape and immune cell responses in the tumor microenvironment. Early work in the MMTV-PyMT transgenic model, harboring a homozygous null mutation for the gene encoding the macrophage growth factor, colony-stimulating factor-1 (CSF-1), demonstrated that macrophages were necessary for metastatic progression *in vivo* (96). As mentioned above, tumor-associated macrophages play multiple roles in promoting cancer metastasis by secreting epidermal growth factor (EGF) to promote motility, invasion, and ECM degradation by cancer cells (97). Such models have additionally implicated adaptive immune cells, IL-4 expressing CD4+ T lymphocytes, in indirectly promoting invasion and metastasis by regulating the phenotype and effector function of CD11b+Gr1-F4/80+ macrophages, ultimately modulating EGF signaling within the cancer cells (98). Other murine models like the K14cre;Cdh1f/f;Trp53f/f (KEP) model further highlighted the importance of immune cell function in tumor progression by demonstrating a role for neutrophil expansion during tumor progression by a γδT cell/IL-17/neutrophil axis (99). Separately, in the syngeneic *TP53* null transplant model of mammary tumorigenesis, the dichotomous distribution of macrophages and neutrophils in murine tumor models was identified, further emphasizing the need for improved characterization of inter-patient heterogeneity of the myeloid compartment (100). At present, TNBC represents the most promising candidate for ICIs given the presence of immune cell infiltrates in subsets of these patients and a higher somatic mutation burden relative to non-TNBC. More recently, the utilization of “mutagenized” tumors by overexpression of the APOBEC3B enzyme in credentialed GEMMs further demonstrated the utility of mouse models in the identification of mechanisms of response to ICI therapy involving B cells and CD4+ T follicular helper cells (101). Future studies using relevant mouse models will be imperative to uncover the spatiotemporal exchanges between cancer and immune cells across both the primary metastatic cellular landscape in an effort to effectively develop novel immunotherapeutic approaches for advanced-stage breast cancers.

## Standard of Care Therapy Resistance in Breast Cancer

Although SOC regimens vary amongst the major breast cancer subtypes, therapeutic resistance is a major clinical issue in each. The foremost classes of targeted therapy used in ER+ breast cancer are selective estrogen receptor modulators (SERMs; *e.g.* tamoxifen), selective estrogen degraders (SERDs; *e.g. fulvestrant)* or aromatase inhibitors (102). SOC for HER2+ breast cancers include anti-HER agents such as small molecule inhibitors, HER2 blocking antibodies, or HER2 antibody drug conjugates (ADCs). As TNBC lacks these cell surface proteins, SOC agents in this setting are currently limited to cytotoxic chemotherapies. In the case of BRCA1/2 deleterious mutant carriers, patients who are often triple negative, PARP inhibitors are currently approved for use in the metastatic setting and are under investigation for use in the neoadjuvant setting.

Mechanisms of therapy resistance can be categorized as: 1) acquired either permanently or reversibly, and either clonally or sub-clonally, following treatment, or 2) pre-existing clonally or sub-clonally prior to treatment. Acquired or pre-existing resistance can be mediated by genomic events (mutations, copy number alterations, genomic structural variants), transcriptional programs, epigenetic modification of chromatin, post-transcriptional regulation of RNA and/or protein levels, and metabolic rewiring. Reversible resistance is often referred to as drug-tolerance or adaptation of “persister” cell phenotypes. These molecular changes can ultimately mediate resistance by enhancing efflux or breakdown of drugs, blocking drug uptake, inhibiting drug-mediated apoptosis, adaptive programs of repair and survival, or protection of CSC features. Tumor cell extrinsic mechanisms driving resistance such as immune system escape, have also been identified. Furthermore, tumor cell dormancy has been found to contribute to therapy resistance, especially in the ER+ subtype with characteristically late-arising metastatic/therapy resistant relapses. In contrast, TNBCs typically exhibit relapses on the scale of only a few years after diagnosis (103). Here we discuss the variety of experimental models that have been used to gain insights into breast cancer therapy resistance.

## *In Vitro* Models of Therapy Resistance—2D

Established breast cancer cell lines provide a tractable platform with which to functionally dissect the roles of putative drivers of resistance discovered by profiling patients’ biopsies. Although these models lack the often important microenvironmental cues of *in vivo* systems, they have provided valuable insights about the biology of breast cancer resistance. While systematic analyses of SOC therapy resistance mechanisms across a multitude of models within each major breast cancer subtype are yet incomplete, some studies have provided snapshots of these mechanisms in defined contexts as described below (**Figure 1**, **Table 1**).

### Drug Tolerant States, Epigenetic Phenotypes, and Metabolic Rewiring

Breast cancer cell lines offer the opportunity to study intra-tumoral heterogeneity and cellular plasticity as they pertain to therapeutic resistance. In an effort to investigate targeted therapies not yet approved as SOC for breast cancers, modeling of the “drug tolerant persister” (DTP) cell subpopulation in basal-like breast cancer cell lines after acute treatment with therapies such as MEK or BRAF inhibitors revealed that epigenetic plasticity, rather than Darwinian selection, was associated with resistance. This study demonstrated that targeting this epigenetic plasticity with a BET inhibitor abrogated the DTP state and cell survival (104). Acute treatment of a broad panel of cancer cell lines, including HER2-positive breast cancer cell lines, with tyrosine kinase targeted inhibitors revealed chromatin modification-mediated adaptation of the DTP state is a common feature of cancer cells (105). A study of ER+ breast cancer cell lines revealed the histone demethylase KDM5 contributed to fulvestrant resistance. KDM5 was found to drive transcriptomic intra-tumor heterogeneity as evidenced by single cell RNA sequencing of cell lines. Single cell analyses and cellular barcode-mediated lineage tracing revealed that the fulvestrant-resistant phenotype pre-existed in a low-abundance genomic subclone prior to treatment of cell lines (106). Furthermore, cell line-based studies of resistance to experimental epigenetic-targeted therapies such as BET bromodomain inhibitors have revealed potential synergistic drug combinations that may prove useful clinically in the future (107, 108).

Treatment of TNBC cell lines with SOC chemotherapy was found to result in adaptation of a polyploid “giant cell” phenotype, a morphological feature that has been observed in chemotherapy-treated human breast tumors (109). These resistant cell lines were characterized by metabolic reprogramming that may provide novel therapeutic opportunities for treating chemoresistant TNBCs. Other studies of acute chemotherapy treatment of MCF7 cells revealed increased expression of proteins related to apoptosis signaling and redox homeostasis (110). Serial analyses of pre- and post-chemotherapy TNBC biopsies has nominated putative drivers and suppressors of adaptive survival programs in post-chemotherapy residual disease. Functionalization of the putative resistance drivers MYC and MCL1 in TNBC cell lines revealed they mediated CSC features through rewiring of mitochondrial oxidative phosphorylation (111). Conversely, the putative resistance suppressor DUSP4 was found to be silenced in post-chemotherapy TNBCs, thus removing its inhibition of ERK signaling (112). Taken together, these studies revealed that breast cancer cell lines can model dynamic, reversible mechanisms of SOC therapy resistance. It is possible these epigenetic mechanisms of therapy resistance are prominent in the context of TNBC due to the lack of a unifying oncogenic driver in this subtype.

### ER and HER2 Pathway Resistance Mechanisms

Numerous ESR1 mutations and gene fusions, reviewed recently (113), have been identified in patient tumor sequencing data associated with resistance and relapse in HR+ positive breast cancers. Many of these mutations have been introduced into breast cancer cell lines for mechanistic studies. For example, the K303R ESR1 mutation, identified in patient tumor sequencing data and ectopically expressed in the MCF7 ER+ cell line, was demonstrated to confer aromatase inhibitor resistance through increased downstream PI3K and IGF1R pathway activation (114, 115). Recurrent ESR1 activating mutations, such as Y537S, Y537N, and D538G, and gene fusions such as ESR1-YAP1 and ESR1-PCDH11X, frequently identified in metastatic ER+ breast cancers, have been introduced into breast cancer cell lines to reveal their role in driving SERM and SERD resistance and to identify collateral lethalities associated with these frequently observed mutations (4–7). ESR1 mutations associated with resistance in breast cancer patients have also been found to naturally occur in ER+ breast cancer cell lines grown under long-term estrogen deprivation (LTED). These LTED cell lines eventually resume proliferation in the absence of estrogen supplementation and were found to harbor a subclonal Y537C mutation (116). Thus, cell lines naturally evolving estrogen-independent growth mechanisms provide an additional system with which to study ESR1 biology. Recently, loss of neurofibromin (NF1), identified in breast cancer patient sequencing data as associated with poor outcomes, was demonstrated in ER+ breast cancer cell lines to function as a transcriptional co-repressor of ER. These findings were then translated *in vivo* using cell line xenografts and PDXs, enabling preclinical trials demonstrating novel therapeutic combinations to treat NF1-low ER+ breast tumors (117).

Anti-HER2 therapy resistance mechanisms include genetic alteration of HER2 itself, reactivation of downstream HER2 signaling, or activation of compensatory pathways (118). These mechanisms have been investigated in a multitude of HER2-positive breast cancer cell lines. For example, long-term exposure of HER2+ cell lines to anti-HER2 drugs revealed that resistance could be conferred through upregulation of ER signaling (119). Xenograftment of HER2-amplified cell lines or ER+ cell lines genetically engineered to over-express HER2 has provided a platform with which to compare the efficacies of anti-HER2 agents in combination with anti-estrogen and targeted therapies (120, 121). Recent studies of HER2+ cell lines and genetically engineered mouse models (MMTV-rtTA/HER2) revealed HER2 therapy resistance can be mediated by cyclin D1/CDK4 and EGFR signaling, providing promising therapeutic targets to overcome resistance that are currently in clinical testing (122).

### Cancer Stem-Like Cells and Drug Efflux

Numerous studies have demonstrated a critical role for CSCs or tumor-initiating cells (TICs) in driving breast tumorigenesis, resistance, and metastasis. These cells can be distinguished from the non-TIC population based on cell surface marker expression (123) and have been identified in human tumors, breast cancer cell lines, GEMMs, and PDX models. Studies in breast cancer cell lines have demonstrated that following exposure to SOC chemotherapies, CSC, TIC, and EMT features and functions can be elevated in cells of the various major subtypes of breast cancer (124–127). These models have provided a robust platform with which to characterize and target transcriptional and signaling regulators of CSC features. Furthermore, breast cancer cell lines with mesenchymal properties were found to exhibit more chemoresistance than were epithelial-like or “hybrid EMT” breast cancer cells (128). As opposed to administering chemotherapies to breast cancer cells grown on plastic, HER2+ breast cancer cells have been xenografted into immune-compromised mice which were then treated with chemotherapy. *Ex vivo* analyses of cells derived from those tumors revealed that chemotherapy exposure *in vivo* had enriched for CSC/TIC features that were maintained in cultures derived from those tumors (129).

Subsets of breast CSCs, termed the “side population”, have been identified that have high expression of drug efflux proteins and are resistant to chemotherapeutics due to their ability to expel drugs from within the cells. This population has been observed in breast cancer cell lines (130). Breast cancer cell lines were used to determine that ROR1, an upstream regulator of the drug efflux pump ABCB1, contributes to chemotherapy resistance and is correlated with CSC features and poor therapeutic responses (131). Importantly, the CSC and drug efflux features of breast cancer *in vitro* models have also been observed in biopsies obtained directly from patients. Development of anti-CSC therapies is a major topic of current investigation in the field and is expected to perturb both therapy resistance and metastasis.

### *In Vitro* Models of Therapy Resistance—3D

Recent advances in 3D organoid culturing methodologies have revolutionized the ability to test SOC and investigational agents in patient- and PDX-derived cells. A major advantage of these organoid models is the relatively low cost and high efficiency when compared with mouse PDX establishment. A biobank of 95 patient-derived primary and metastatic breast cancer organoids was recently described that preserves many of the histologic and genomic features of donor patient’s tumors. These organoids were leveraged for high-throughput drug screening. Interestingly, direct comparison of tamoxifen response in patients with their matched organoid cultures revealed congruent responses (14). Similarly, organoids have been derived from orthotopic PDX models, enabling high-throughput drug screening with panels of SOC and experimental compounds, providing novel avenues for preclinical drug testing (132) and synergistic combinations (133). Direct genomic and pharmacologic comparisons of organoids *in vitro* and tumors derived from orthotopic xenotransplantation into mice has revealed a high degree of concordance (16). Together, these studies reveal that patient- and PDX-derived organoid cultures are promising platform with which to efficiently and speedily test the efficacies of SOC and investigational therapies for clinical translation. There is a great deal of excitement that the relative speed and ease of investigational drug testing in patient-derived organoid cultures, when compared with establishment of PDX mice, will finally enable rapid, real-time, implementation of personalized therapies tailored for patients exhibiting resistance to SOC therapies.

### *In Vivo* Cell Line Xenograft PDX Models of Therapy Resistance

PDX models enable experimentation with minimally manipulated human tumor cells in an organismal microenvironment, one that albeit lacks a fully functional immune system. Several studies have utilized these models to study SOC therapy resistance, revealing novel biological insights and trends matching those observed in patients’ tumors. These models also afford the ability to study the conjoined phenotypes of metastasis and therapy resistance, which often co-occur in models and in patients. Two main approaches have been used with these models: 1) discovery-based approaches in which SOC agents are administered to PDXs, then tumors are sampled longitudinally to identify mechanisms of resistance, and 2) preclinical testing approaches monitoring the efficacy of experimental agents or combinations with SOC.

As an example of a discovery approach, treatment of TNBC PDX models with standard front-line chemotherapies revealed diverse responses across models derived from distinct patients. A subset of models harbored resistance accompanied by a reversible drug-tolerant phenotypic state in the absence of clonal selection. Lentiviral barcode-mediated clonal tracking in these models enabled monitoring of clonal architecture throughout treatment *in vivo* and, combined with transcriptomic profiling, revealed targeted therapy options that were translated into preclinical trials in PDXs (134). Studies such as these have revealed novel therapeutic avenues such as oxidative phosphorylation inhibition in the case of TNBC (134, 135). A longitudinal profiling study of long-term single-agent taxane treatment of TNBC PDX models delineated dynamic maintenance of TIC populations as resistance arose (136). A study of BRCA1-deficient PDX models was conducted to longitudinally characterize resistance to SOC chemotherapies and PARP inhibitors. This study identified previously known, as well as novel, mechanisms of BRCA1 reactivation, including *de novo* gene fusion events (137). In each of these studies, aspects of these resistance mechanisms were validated in unmanipulated patients’ biopsies, revealing that PDX models are effective tools with which to discover *bona fide* resistance drivers with clinical relevance.

In the second type of approach, PDX models have also proven a robust platform with which to test the efficacy of experimental and repurposed anticancer drugs, such as BET bromodomain inhibitors in TNBC (138). In the HER2+ breast cancer setting, PDXs were instrumental in demonstrating the efficacy of CDK4/6 inhibition in overcoming anti-HER2 therapy resistance (122). As discussed above, ESR1 mutations contribute to therapy resistance and metastasis in ER+ breast cancers. PDX models bearing naturally occurring ESR1 mutations have been valuable tools with which to test endocrine therapies (5) and targeted inhibitors against oncogenic kinases such as RON to overcome endocrine therapy resistance (139). Furthermore, use of PDX models affords the capacity to test the efficacy of stroma-targeted therapies such as anti-angiogenesis agents (140) and endothelium-targeted chimeric antigen receptor T cells (141). As these models lack an intact immune system, most PDX studies to date have focused on tumor cell-intrinsic mechanisms of resistance. It will be of vital importance to expand these studies to PDX models with ‘humanized’ immune system components as those technologies evolve in the future.

## *In Vivo* GEMMs of Therapy Resistance

Preclinical GEMMs, in addition to their ability to model several aspects of tumor progression, can be leveraged to provide insights into the mechanisms of therapy response and resistance. One such model recapitulated *BRCA1*-mutated breast cancer by means of *K14Cre;Brca1fl/fl;Trp53fl/fl (*KBIP) genetics. In particular, these tumors exhibited a hypersensitivity to platinum drugs and PARP inhibitors, yet like patients, GEMMs succumbed to acquired resistance (142, 143). These tumors up-regulated drug efflux transporters and homologous recombination. GEMMs have also enabled the identification of several other mechanisms of therapeutic resistance, involving a stroma-related gene signature as a predictor of resistance to neoadjuvant chemotherapy (144, 145), stromal-derived exosome uptake as a determinant of radiation- and chemotherapy-resistance (146), and tumor-associated fibroblast promotion of Her2-targeted resistance through FGFR2 (147). Given the accurate reflection of breast cancer subtypes by GEMMs, the testing of new drugs, combinations, and schedules can be evaluated in such models to provide predictive value for patients (148). Much like the isolation and selection of metastatic derivatives, GEMMs can be used to serially expand therapeutically resistant tumors, propagate them, and then test and screen for therapeutic vulnerabilities in the resistant setting (149). Relative to PDX models, lower cost is a significant advantage to the use of GEMMs; however, they only represent surrogates to their patient counterparts and do not always reflect the complex genomic intra-tumor heterogeneity observed in breast cancer patients’ tumors.

## Tumor Dormancy and Microenvironmental Impacts on Therapy Resistance

As described above, tumor cell dormancy in the context of DTCs that have seeded at metastatic sites but not yet outgrown, is a major issue due to their ability to evade therapeutic treatment and their long-term survivability (27). DTCs have been found to persist at metastatic sites, often undetected by standard clinical means, for many years and are thought to lead to the often-late relapses observed in ER+ cancers. Available models to study metastatic dormancy were recently reviewed (150). DTCs were identified in the bone marrow of Balb/c immune-competent mice following orthotopic implantation of mouse mammary tumor 4T1 cells and surgical resection of primary tumors. These DTCs were shielded from killing by standard cytotoxic chemotherapies by the bone marrow microenvironment (specifically, the vascular endothelium). Therapeutic inhibition of the interaction between DTCs and the endothelium prevented eventual bone metastasis in these models (28). Numerous studies describing the role of tumor cell dormancy in therapy resistance have been reviewed recently (151). For example, in ER+ breast cancer cells made resistant to endocrine therapy, dormancy gene expression signatures were identified by single cell RNA sequencing (152). Furthermore, *in vitro* dormancy models have been used to demonstrate bone marrow secreted factors are able to induce ‘re-awakening’ (*i.e.* growth) of dormant ER+ breast cancer cells (153).

The contribution of stroma to therapy resistance is also an active area of investigation, especially leveraging *in vivo* models comprising stromal compartments. For example, analysis of BRCA mutant TNBCs unexpectedly revealed extensive macrophage infiltration in this subtype. Use of *ex vivo* macrophage cultures, PARP-deficient GEMMs, and BRCA-deficient xenografts revealed that PARP1 aides in macrophage development and that combination of a PARP inhibitor with a CSF1 receptor-blocking antibody enhanced tumor responses in the BRCA-mutant setting (154). Numerous studies using *in vitro* and xenograft models have also revealed a functional role for cancer-associated fibroblasts in SOC therapy resistance in breast cancers (155), as recently reviewed (156). Studies such as these have clearly demonstrated that the roles of dormancy, therapy resistance, microenvironmental crosstalk, and metastasis are closely intertwined.

## Functional Genomics Screens for Mediators of Breast Cancer Resistance

Genome-wide shRNA screening in breast cancer cell lines has enabled high-throughput identification of genes required for cell viability in the context of various oncogenic drivers and have informed synergistic drug combinations (157, 158). Leveraging shRNA screens in defined genetic backgrounds of well characterized cell lines, such as in the context of PTEN-null lines, has enabled identification of vulnerabilities relevant to genetic driver events recurrent in breast cancer patient populations (159). Knock-down screens in the context of SOC therapeutic treatment are only beginning to be adopted and can provide insights into functional mediators of therapy resistance. A barcoded RNAi screen in a HER2 positive cell line revealed trastuzumab resistance could be conferred only by PTEN loss out of a library targeting approximately 8,000 genes. The importance of this pathway was corroborated by the finding that PIK3CA oncogenic mutations similarly conferred resistance to trastuzumab (160). A study conducting genome-wide shRNA screens in 77 breast cancer cell lines revealed functional vulnerabilities of breast cancer cells *en masse*. When compared with high-throughput drug screening data generated in these lines, cross-referencing gene essentiality with drug resistance data in cell lines yielded valuable insights into putative mediators of drug resistance (161). Future expanded application of screening methodologies in the context of therapeutic treatments in breast cancer cell lines and organoids is expected to reveal valuable biological insights and potential therapeutic combinations.

*In vivo* functional genomics screens hold further promise to yield clinically relevant insights into mediators of therapy resistance. Several groups have leveraged high-throughput shRNA or CRISPR/Cas9 libraries subsequently xenografted into immune-compromised mice in other cancer contexts (162, 163). These technologies are only beginning to be leveraged in breast cancer models and have not been applied to the issue of SOC therapy resistance as of yet. One recent study revealed genes required for *in vivo* tumorigenic capacity in subcutaneously xenografted TNBC cell lines, revealing genes involved in CSC feature maintenance (164). A unique screening strategy was used to identify tumor cell genes involved in immune-microenvironment communication. A murine TNBC cell line was transduced with a genome-wide shRNA library, then subcutaneously transplanted into immune-competent and immune-compromised mice. This novel screening approach revealed several genes that were functionally validated to mediate *in vivo* sensitivity to immune recognition, providing potential targets for future immune therapies (165). *In vivo* screening is limited by library complexity achievable in tumor models, as well as cost of animal acquisition and maintenance. However, application of shRNA and CRISPR/Cas9 libraries in orthotopically xenografted breast cancer cell lines and PDX models, as well as genetically engineered mouse models, upon treatment with SOC therapies is expected to provide invaluable insights into clinically relevant functional drivers of resistance in breast cancer.

## Concluding Remarks

Therapy resistance and metastasis continue to be the two major causes of breast cancer mortality. The research works reviewed herein have provided valuable insights into mechanisms driving metastatic recurrence and treatment resistance. Continued advancements in the field are needed to push scientific boundaries to provide comprehensive insights into clinically relevant mechanisms of cancer relapse. Additionally, as therapies generate alterations in the tumor biology, modeling appropriate disease outcomes will be imperative in order to accurately predict metastatic behaviors. Acquisition, expansion, and ease-of-use of PDX models with ‘humanized’ microenvironmental components is expected to revolutionize the field. Use of these humanized PDX models for gene and protein expression profiling, lineage tracing, clonal tracking, comparison of multi-site metastases, longitudinal profiling throughout therapeutic treatment, and high-throughput ORF and CRISPR/Cas9 screening are expected to provide unprecedented biological insights. By including a more physiologically relevant immune system, results from these studies may be more readily translatable to the clinic. Moreover, in vitro 3D organoid applications composed of multi-component platforms that recapitulate an appropriate tumor microenvironment will provide the ability to experimentally interrogate meaningful cell and biological interactions driving disease progression and could theoretically provide real-time personalized therapeutic information for patients. As laboratory and clinical research progress, the next generation of therapies will become the new “standard of care”. As these develop, novel mechanisms of resistance to those agents should be anticipated and deeply investigated in the laboratory. With useful models, the mysteries of metastasis and recurrence will gradually be unraveled with time.

## Author Contributions

KR and GE conceived of and wrote the manuscript. All authors contributed to the article and approved the submitted version.

## Funding

The authors are supported by a CPRIT faculty recruitment award RR200009 to GE, National Institutes of Health (NIH) 1K22CA241113-01 to GE and 5K22CA207463 to KR, and Susan G. Komen CCR18548284 to KR. The content is solely the responsibility of the authors and does not necessarily represent the official views of NIH, Susan G. Komen, or CPRIT.

## Conflict of Interest

The authors declare that the research was conducted in the absence of any commercial or financial relationships that could be construed as a potential conflict of interest.
